# Effects of conjugated linoleic acid and exercise on bone mass in young male Balb/C mice

**DOI:** 10.1186/1476-511X-5-7

**Published:** 2006-03-23

**Authors:** Jameela Banu, Arunabh Bhattacharya, Mizanur Rahman, Marianne O'Shea, Gabriel Fernandes

**Affiliations:** 1 Department of Medicine, Division of Clinical Immunology and Rheumatology, University of Texas Health Science Center at San Antonio, 7703, Floyd Curl Dr, San Antonio, 78229-3900, USA; 2 Loders Croklaan Lipid Nutrition, Minneapolis, MN 55410, USA

## Abstract

There is an increase in obesity among the population of industrialized countries, and dietary supplementation with Conjugated Linoleic Acid (CLA) has been reported to lower body fat mass. However, weight loss is generally associated with negative effects on bone mass, but CLA is reported to have beneficial effects on bone. Furthermore, another factor that is well established to have a beneficial effect on bone is exercise (EX). However, a combination therapy of CLA and EX on bone health has not been studied. In this paper, we report the beneficial effects of CLA and EX on bone, in four different groups of Balb-C young, male mice. There were 4 groups in our study: 1. Safflower oil (SFO) sedentary (SED); 2. SFO EX; 3. CLA SED; 4. CLA EX. Two months old mice, under their respective treatment regimens were followed for 14 weeks. Mice were scanned *in vivo *using a DEXA scanner before and after treatment. At the end of the treatment period, the animals were sacrificed, the left tibia was removed and scanned using peripheral quantitative computerized tomography (pQCT). The results showed that although CLA decreased gain in body weight by 35%, it however increased bone mass by both reducing bone resorption and increasing bone formation. EX also decreased gain in body weight by 21% and increased bone mass; but a combination of CLA and EX, however, did not show any further increase in bone mass. In conclusion, CLA increases bone mass in both cancellous and cortical bones, and the effects of CLA on bone is not further improved by EX in pure cortical bone of young male mice.

## Background

Obesity is a major health disorder in the US. About 30.5% of the adult population in the US is obese [1]. In the last decade alone the percent of obese people has doubled according to the National Health and Nutrition Examination survey [1]. Body Mass Index (BMI) is the tool currently used to measure excess body weight and BMI of 30 and above represents obesity. The influence of body weight on bone mass has been extensively studied [2-6]. Increase in body weight increases bone mass and reduction in body weight decreases bone mineral density (BMD) [2,4-6]. Since obesity is a growing problem, many people are turning towards agents that will reduce body fat mass. Recently, conjugated linoleic acid (CLA) has been reported to have significant effect on lowering body fat mass [7]. CLA is a group of fatty acids that has a mixture of positional and geometric isomers of linoleic acid with conjugated double bonds, which may be of *cis *and *trans *configuration at positions 9 and 11 or 10 and 12 [8,9]. Several reports have brought to light the beneficial effects of dietary CLA supplementation in experimental animals and humans [9-12]. CLA not only reduces body fat, but also reduces inflammation, has anti carcinogenic properties, and enhances bone mineralization [13-15]. Several *in vitro *and *in vivo *studies have also shown that CLA has positive influence on bone [12,16,17].

Increase in body weight increases bone mass but aging is associated with bone loss in both men and women [18]. Bone loss causes thinning of bones thereby increasing the risk of fractures [19]. In the last decade, more evidence has suggested that males also lose almost the same amount of bone as women [20]. The major difference between bone loss in women and men is that bone loss in women is severe during and after menopause, while bone loss in men is very gradual. Every individual loses 0.5–1% of bone every year, for the rest of their life, after attaining peak bone mass around their third decade of life [21]. One of every four men, above the age of 50, is susceptible to osteoporosis related fracture. Elderly men, when they have fractures are more disabled. Although, several therapies are available for the treatment of osteoporosis, the risk of side-effects and/or lack of increasing bone anabolic agents have increased the need for alternate therapies. Dietary supplementation of beneficial compounds is encouraged in order to reduce the risk of side-effects. Since CLA is reported to reduce both body fat as well as bone loss, it was our interest to study the effects of CLA on bone mass.

It is very well known that bone formation and resorption is also influenced by exercise (EX) [22,23]. Exercise exerts strain on bone, which stimulates bone formation and decreases bone resorption. According to the 'Mechanostat theory [24] bone is maintained by a feedback mechanism such that mechanical strains do not exceed a minimum effective strain. There are several reports indicating that exercise increases bone formation [25-28].

In this paper, we investigated whether a significant loss of body weight and particularly, combined effects of CLA and exercise on loss of body weight will have any adverse or beneficial effects on bone mass in young male Balb/C using Dual Energy X-ray Absorptiometry (DEXA) and Peripheral Quantitative Computerized Tomography (pQCT) densitometry. The results show that both CLA and exercise do not cause any adverse effects but appear to protect against bone loss during weight loss caused by CLA and exercise.

## Results

### Body weights

**Figure 1:**
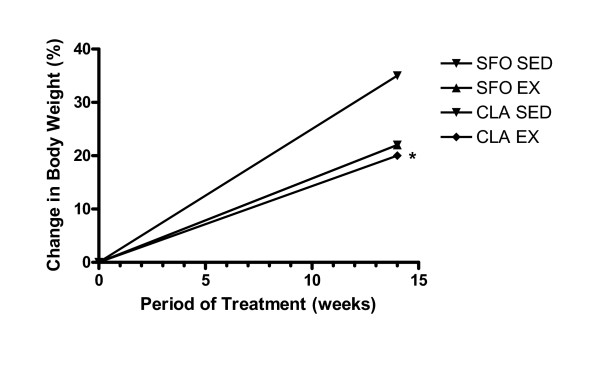
Percent change in body weights after the different treatment regimens. * p < 0.01 vs SFO SED.  SFO SED = Safflower Oil (SFO) Sedentary (SED);  SFO EX = SFO and Exercise (EX);CLA SED = Conjugated linoleic acid (CLA) SED; and  CLA EX = CLA and EX.

Mice that were fed CLA had significantly lower gain in body weight during the treatment period. Figure 1 shows the change in percent body weight in mice fed the different diets. CLA SED treated mice showed a 35% (P < 0.01) decrease in body weight gain when compared to that of SFO SED treated mice. As expected mice from both EX groups also had lower weight gain (21%).

### DEXA

#### Effects of CLA

CLA significantly increased the BMD by 15% (p < 0.05), 12% (p < 0.05), 8% (p < 0.05), 25% (p < 0.05) and 26% (p < 0.05) in the DFM, FD, TD, L3 and L5 respectively, when compared to that of SFO SED mice (Table 2).

**Table 1:** Composition of semi-purified AIN-93M diet.

**Ingredient, g/Kg**	**SFO**	**CLA**
Casein	248	248
Salt mix	64	64
Vitamin mix	17	17
Cellulose	55	55
Cerelose	405.5	403.74
Choline bitartrate	2.5	2.5
dl-methionine	3	3
Clarinol powder*	0	6.76
SFO	5	0
Corn oil	200	200
Total	1000	1000

*contains 74% CLA.

SFO = Safflower Oil.

CLA = Conjugated linoleic acid

**Table 2:** Effects of CLA and EX on BMD (g/cm^2^) from different bone sites analyzed using DEXA densitometry.

**Groups/Bone sites**	**SFO SED**	**SFO EX**	**CLA SED**	**CLA EX**
**Distal Femoral Metaphysis**	0.3355 ± 0.0200^a^	0.3368 ± 0.0010^b^	0.3654 ± 0.0001	0.3654 ± 0.0001
**Proximal tibial Metaphysis**	0.2582 ± 0.0080^b^	0.2846 ± 0.0060^a^	0.3053 ± 0.0050	0.2846 ± 0.0060^a^
**Femoral Diaphysis**	0.3341 ± 0.0100^a,b,c^	0.3730 ± 0.0008	0.4059 ± 0.0100	0.3767 ± 0.0009
**Tibial Diaphysis**	0.1994 ± 0.0008^a^	0.1944 ± 0.0006^a,b^	0.2107 ± 0.0003	0.2107 ± 0.0003
**Lumbar Vertebra (L3)**	0.1956 ± 0.0010^a^	0.2107 ± 0.0010	0.2449 ± 0.0100	0.2244 ± 0.0008
**Lumbar Vertebra (L4)**	0.2289 ± 0.0006	0.2572 ± 0.0200	0.2515 ± 0.0010	0.2515 ± 0.0010
**Lumbar Vertebra (L5)**	0.2076 ± 0.0009^a,b^	0.2618 ± 0.0006	0.2696 ± 0.0150	0.2696 ± 0.0150

a = p < 0.05 vs CLA SED, b = p < 0.05 vs CLA EX, c = p < 0.05 vs SFO EX. SFO SED = Safflower Oil (SFO) Sedentary (SED), SFO EX = SFO and Exercise (EX), CLA SED = Conjugated linoleic acid (CLA) SED, CLA EX = CLA and EX.

#### Effects of EX

EX significantly increased the BMD by 13% (p < 0.05) in the FD of SFO EX treated mice when compared to that of SFO SED treated mice. In DFM and lumbar vertebrae also had higher BMD but this increase was not statistically significant (Table 2).

#### Combined effects of CLA and EX

The BMD values at the different bone sites studied were found to be lower than that of the CLA SED treated mice. This decrease was not statistically significant except in the PTM were a decrease of 7% (p < 0.05) was observed when compared to that of CLA SED treated mice (Table 2).

When compared to SFO EX treated mice there was significant increase in BMD only in the PTM (10%, p < 0.05) and TD (8%, p < 0.05). In all other regions studied, the BMD increased, but this increase was not statistically significant (Table 2).

### pQCT densitometry

#### PTM

##### Effects of CLA

CLA increased Cn. B. Ar. (24%, p < 0.05), Cn. BMD (46%, p < 0.05), Ct. BMC (24%, p < 0.05), Ct. BMD (17%, p < 0.05), Peri. PM (18%, p < 0.05) and Endo PM (21%, p < 0.05) when compared to those of SFO SED treated mice (Table 3).

**Table 3:** Effects of CLA and EX on the Cancellous and Cortical Bone parameters of the Proximal Tibial Metaphysis Analyzed using pQCT densitometry.

**Groups/Bone Parameters**	**SFO SED**	**SFO EX**	**CLA SED**	**CLA EX**
Cancellous Bone Area (mm^2^)	4.127 ± 0.200^a^	4.020 ± 0.400	5.110 ± 0.300	3.890 ± 0.400
Cancellous BMC (mg/mm)	6.893 ± 0.039	6.668 ± 0.058	7.951 ± 0.071	6.663 ± 0.055
Cancellous BMD (g/mm^3^)	494.6 ± 20.4^a^	539.9 ± 29.4	721.8 ± 96.1	553.3 ± 39.4
Cortical Bone Area (mm^2^)	3.749 ± 0.170	3.778 ± 0.270	4.469 ± 0.200	4.055 ± 0.267
Cortical BMC (mg/mm)	3.424 ± 0.180^a^	3.499 ± 0.240	4.260 ± 0.190	3.972 ± 0.260
Cortical BMD (g/mm^3^)	2726.0 ± 80.5^a,b^	2813.0 ± 64.5^a,b^	3181.0 ± 93.4	3025.0 ± 50.0
Cortical Thickness (mm)	0.759 ± 0.040	0.712 ± 0.040	0.808 ± 0.055	0.799 ± 0.040
Periosteal Perimeter (mm)	18.20 ± 0.70^a^	17.96 ± 1.10	21.44 ± 0.74	16.60 ± 2.10
Endocortical Perimeter (mm)	13.62 ± 0.49^a^	13.42 ± 1.05	16.44 ± 1.00	12.28 ± 1.40^a^

a = p < 0.05 vs CLA SED, b = p < 0.05 vs CLA EX. SFO SED = Safflower Oil (SFO) Sedentary (SED), SFO EX = SFO and Exercise (EX), CLA SED = Conjugated linoleic acid (CLA) SED, CLA EX = CLA and EX.

##### Effects of EX

Although EX did not bring about any significant changes in the different parameters studied, SFO EX treated mice showed higher Cn. BMD and Ct. BMD values (Table 3).

##### Combined effects of CLA and EX

Slight decrease was observed in all the different parameters studied in the CLA EX treated mice when compared to those of the CLA SED treated mice. These decreases were not statistically significant except in the Endo PM where 34% (p < 0.05) decrease was observed. (Table 3). Ct. BMD increased by 8% (p < 0.05) in CLA EX treated mice when compared to that of SFO EX treated mice.

### TF junction

#### Effects of CLA

CLA increased Ct. BMC, Ct. BMD, Ct. Th, Peri PM and Endo PM significantly by 29% (p < 0.05), 17% (p < 0.05), 4% (p < 0.05), 25% (p < 0.05) and 19% (p < 0.05) when compared to those of SFO SED treated mice. The Ct. B. Ar also increased but this increase was not statistically significant (Table 4).

**Table 4:** Effects of CLA and EX on the Cortical Bone Parameters of the Tibia Fibular Junction Analyzed by pQCT densitometry.

**Groups/Bone Parameters**	**SFO SED**	**SFO EX**	**CLA SED**	**CLA EX**
**Cortical Bone Area (mm^2^)**	2.779 ± 0.350	2.857 ± 0.120	3.347 ± 0.110	2.995 ± 0.060^a^
**Cortical BMC (mg/mm)**	3.303 ± 0.400^a^	3.672 ± 0.200	4.248 ± 0.070	3.811 ± 0.070^a^
**Cortical BMD (g/mm^3^)**	3647.0 ± 171.3^a^	3897.0 ± 112.4	4281.0 ± 84.7	3915.0 ± 106.6^a^
**Cortical Thickness (mm)**	0.873 ± 0.057^a^	0.957 ± 0.030	1.044 ± 0.020	0.961 ± 0.020^a^
**Periosteal Perimeter (mm)**	11.36 ± 0.89^a^	12.07 ± 0.43	14.24 ± 0.37	12.58 ± 0.26^a^
**Endocortical Perimeter (mm)**	5.746 ± 0.430^a^	6.809 ± 0.310	6.819 ± 0.148	6.543 ± 0.180

a = p < 0.05 vs CLA SED. SFO SED = Safflower Oil (SFO) Sedentary (SED), SFO EX = SFO and Exercise (EX), CLA SED = Conjugated linoleic acid (CLA) SED, CLA EX = CLA and EX.

#### Effects of EX

EX also increased all the cortical parameters studied in the SFO EX treated mice when compared to those of the SFO SED treated mice, but these increases were not statistically significant (Table 4).

#### Combined effects of CLA and EX

CLA EX decreased the Ct. B. Ar, Ct. BMC, Ct. BMD, Ct. Th and Peri PM by 11% (p < 0.05), 19% (p < 0.05), 9% (p < 0.05), 8% (p < 0.05) and 12% (p < 0.05) respectively, when compared to those of the CLA SED treated mice (Table 4).

However, when compared to SFO EX treated mice, CLA EX treatment increased all the cortical parameters but this increase was not statistically significant (Table 4).

## Discussion

In this study, we observed that there was significant loss of overall body weight and body fat mass in CLA treated mice. The detailed results are published elsewhere [7]. In spite of significant decrease in the gain of body weight in CLA fed mice, BMD in the lumbar vertebrae and PTM increased significantly. In the PTM, where both cancellous and cortical bones are present, CLA increased bone area and BMD in the cancellous bone suggesting that CLA has beneficial effects on cancellous bone. CLA also increased bone mineralization and bone density in the cortical bone surrounding the cancellous bone in the PTM, showing that overall CLA has a positive effect on bone. In pure cortical bone (TF), CLA increased bone mass by increasing the periosteal perimeter. This increase in the periosteal surface suggests that CLA, to some extent, influences bone formation as well. Recently, the effects of CLA on bone have been investigated in a few *in vitro *as well as *in vivo *studies. *In vitro *studies using MC3T3-E1 osteoblast like cells showed that CLA increased levels of osteocalcin, alkaline phosphatase activity and calcium absorption [29]. Calcium absorption is enhanced in the presence of polyunsaturated fatty acids (PUFA) [15]. *In vivo *studies using young chicken, mice and pigs have shown that in the presence of CLA there is generally increased bone mass. However, in young rats, CLA decreased bone formation rates [30,31], but when CLA was given along with n-6 PUFA, bone formation rates increased [32]. In humans, a study that tested the effects of CLA supplementation on the biochemical markers of bone metabolism [33] has reported that in adult men CLA supplementation did not affect markers of bone metabolism. A recent study on the effects of CLA on postmenopausal women has concluded that dietary intake of CLA may benefit BMD [34].

**Figure 2:**
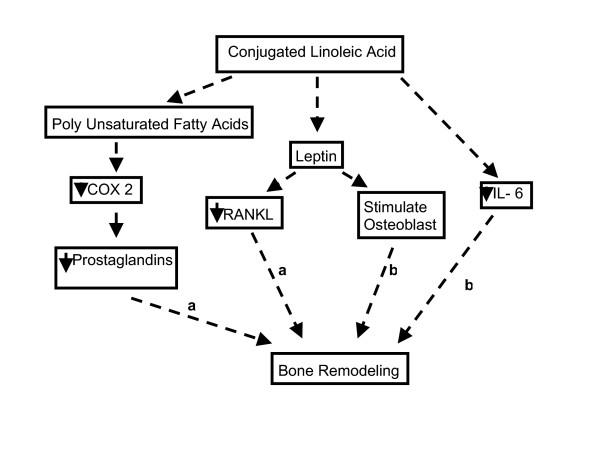
Schematic Representation of the Possible Pathways Involved in the Action of CLA on Bone Mass. a = decrease bone resorption; b = increase bone formation.

The mechanism by which CLA exerts its effect on bone is still not very clear, although a few pathways are reported recently (Fig 2). *In vitro *studies conducted in our lab showed that CLA (1 μM) significantly inhibited osteoclastogenesis (unpublished data). CLA, in the presence of PUFA, modulated the action and expression of COX-2 enzyme, thereby altering prostaglandin-dependent bone resorption [35,36]. CLA also alters leptin concentration due to its action on adipocytes; leptin expression was found to be modulated both in *in vitro *cell cultures as well as in *in vivo *animal studies [37-40]. Indeed, CLA may exert its influence on bone mass through down regulation of leptin [41,42]. Leptin is reported to decrease the production of RANK (Receptor Activated Nuclear Factor) ligand [43], which regulates osteoclast differentiation and activation, thereby influencing bone resorption [44]. There is evidence that leptin also stimulates the formation of osteoblasts [42]. Studies have reported that IL-1 and IL-6 induce bone resorption by stimulating the recruitment and formation of osteoclasts; thereby, releasing calcium from bone [45,46]. Dietary CLA reduces both pro-inflammatory IL-1 [47] and IL-6 production [48] Reduced levels of leptin and IL-6 were observed in serum of mice from the present study, and the results are published elsewhere [7]. The reduction of leptin suggests that CLA may not use the leptin pathway to reduce bone resorption, but the circulating leptin may be sufficient to stimulate osteoblasts and in turn increase bone formation on the periosteal surface. Decrease in IL-6 suggests that CLA may use the IL-6 and RANK-L pathways to reduce bone resorption. We, therefore, believe that CLA may have bone anabolic properties as well as bone anti-resorption properties similar to n-3 fatty acids which was found to protect bone loss in mice prone to develop autoimmune disease [49].

In the present study, EX alone showed a trend towards increasing bone density in PTM and TF junction. Such effects of EX on bone has been reported in several studies. In aged female Sprague Dawley rats, treadmill exercise increased BMD in the tibial metaphysis and L5 vertebrae. In these regions, trabecular separation decreased, and trabecular volume and number increased in exercised rats. This increase in BMD in cancellous bone can be attributed to increased bone formation and reduced bone resorption [28]. In the present study also we found that EX increased bone formation and decreased bone resorption. In male rats, it has been reported that EX increased pure cortical bone area by increasing bone formation [50] similar to the present study, whereas in the TF junction, BMD and bone thickness increased in the exercised mice, mainly, by increasing the periosteal perimeter. EX, in general, brings about site specific responses on bone [23,25]. BMD is increased more in cancellous bone than in pure cortical bone as has been reported earlier in middle-aged Fisher 344 rats [25,26].

In combination, CLA and EX had beneficial effects on lumbar vertebrae and femoral diaphysis. The combination treatment did not show any changes in cancellous bone of the PTM, except that it significantly reduced the endocortical perimeter, suggesting that in combination CLA and EX not only inhibited endosteal bone resorption but also increased endocortical bone formation. In pure cortical bone, however, effects of CLA and EX are moderate; there was a significant decrease in all the cortical parameters studied with respect to CLA SED treated mice, but still higher than the SFO EX treated group. So, CLA with EX has a positive effect on pure cortical bone, but for some reason it is not as beneficial as CLA alone. There maybe several explanations for this. EX by itself, as mentioned earlier, is reported to have site-specific effects on bone and is known to have more effect on cancellous bone than on cortical bone [25,26], similar to what we observed in this study. It is also possible that EX is able to modulate the effects of CLA selectively on cortical bone. Another possibility is that the type and duration of EX used in this study is insufficient to bring about more changes to the cortical bone. Whatever the case may be, the encouraging part is that CLA and EX together is still beneficial to pure cortical bone.

This is the first report on the combined favorable effects of CLA and EX on bone mass in young male mice. Based on our results we have shown that CLA alone is capable of increasing both cancellous and cortical bone mass; although, it effectively reduces body weight in this animal model. It can therefore be suggested that CLA can be used as an efficient supplement for weight management without having any adverse effects on bone. The question whether CLA and EX in combination is more beneficial to bone remains to be clarified by undertaking further studies in young and old mice. Although, in the different bone sites studied, CLA and EX has a positive effect on bone, they do not seem to fully compliment each other. In fact, EX seems to be lowering the effects of CLA on pure cortical bone. The interaction of CLA and EX at the molecular level is not known. Further studies are required in this regard to throw light on how EX modulates the effects of CLA on bone. However, we conclude that in young male mice, although, CLA reduces body weight, it actually protects both cancellous and cortical bone mass and has no adverse effects on bone due to loss of body weight. EX does not seem to compliment, any further, the effects of CLA.

## Methods

### 1. Animals and experimental diets

Six-week-old male Balb/c mice were obtained from Harlan, Indianapolis, Indiana Weight matched mice were housed in laboratory animal care facility in cages (5 mice/cage) and fed standard lab chow diet. At 8 weeks of age, animals were divided into four groups: Group 1. AIN-93M diet with safflower oil (SFO) (0.5%), sedentary (SFO SED) (n = 6); Group 2. AIN-93M diet with safflower oil (0.5%), Exercise (SFO EX) (n = 6); Group 3. AIN-93M diet with CLA (0.5%), sedentary (CLA SED) (n = 6); Group 4. AIN-93M diet with CLA (0.5%), exercise (CLA EX) (n = 5). Each diet contained 20% corn oil (CO) as high fat diet. Groups 3 and 4 received 0.5% CLA (Clarinol ^TM ^Powder, Lipid Nutrition, Channahon, Illinois) (c9t11 and t10c12 isomers in equal proportions together). The composition of AIN-93M diet is modified form of AIN-93 diet and the details are provided in table 1. Fresh diet was prepared weekly, stored in aliquots at -20°C and provided daily *ad libitum*.

Animals were maintained on a 12 hr light/12 hr dark cycle in an ambient temperature of 22–25°C and 45% humidity. Body weight was monitored weekly and food consumption based on residual food was also monitored everyday. Mice were given the respective diets for 14 weeks and at the end of which they were sacrificed. The left tibia was dissected out and stored in 70% ethanol until it was scanned using pQCT. Animals were handled according to NIH guidelines provided under "The guide for the care and use of laboratory animals" and the Institutional Animal Care and Use Committee of the University of Texas Health Science Center at San Antonio. The body weight data has been reported in another publication [7].

### 2. Exercise protocol

Exercise was performed on a rodent treadmill (Quinton Instruments,Seattle) with the following protocol as described by Fernandes et al [51]. After training for several days on a treadmill containing 10 running compartments, each mouse thereafter ran for 40 to 50 minutes, 5 days/week at 0.6 miles/hour and at 10° angle to provide resistance for running. The treadmill consists of rubber belts, which are driven at a controlled speed. Each mouse was trained to recognize two signals: breaking an electric beam (creates noise) and a mild, continuous airflow. The sedentary mice were placed on the stationery rodent treadmill, twice a week, for the same period of time as the exercised groups.

### 3. Measurement of bone mineral density (BMD)

BMD was measured by DEXA using a Lunar Piximus mouse bone densitometer (GE) and data was analyzed with PIXImus software [52-54]. Before scanning was performed, mice were anesthetized with intra-muscular injection of mouse cocktail containing-Ketamine (3 cc), S.A. Rompun (2 cc) and NaCl (5 cc). During the measurements, the mice were lying in a prone position with posterior legs maintained in external rotation with tape. The hip, knee and ankle articulations were in 90° flexion. BMD was obtained for the entire body except the head. BMD was then measured manually at the left distal femoral metaphysis (DFM), the proximal tibial metaphysis (PTM), the tibia fibular junction (TF) and the lumbar vertebra (L3). Baseline scans were first taken before the administration of the diets and scanning was performed in live mice at the end of 14 weeks.

### 4. Peripheral quantitative computerized tomography densitometry (pQCT)

The cortical bone at the tibia fibula junction (TF) and the cancellous and cortical bones at the proximal tibial metaphysis (PTM) were analyzed by pQCT densitometry, using a XCT research M system (Norland Stratec, Birkenfeld, Germany) as described previously [25,26]. In the TF junction, pure cortical bone was scanned and the following parameters determined: cortical bone area (Ct. B. Ar), cortical bone mineral content (Ct. BMC), cortical bone mineral density (Ct. BMD), cortical thickness (Ct. Th), periosteal perimeter (Peri. PM) and endocortical perimeter (Endo. PM). In the PTM, both cancellous bone and cortical bone surrounding the cancellous bone were analyzed, 1 mm distal to the knee joint, and the following parameters determined: cancellous bone area (Cn. B. Ar), cancellous bone mineral content (Cn. BMC), cancellous bone mineral density (Cn. BMD), Ct. B. Ar, Ct. BMC, Ct. BMD, Ct. Th, Peri. PM and Endo. PM.

### 5. Statistical analysis

Results are expressed as Mean ± SE. Data were normalized to body weight and analyzed with one-way ANOVA and unpaired t-test using GraphPad Prism 4 (GraphPad Software Inc, San Diego, CA, USA). p < 0.05 was considered to be significant. Newman-Keuls multiple comparison test was used to analyze the differences between groups for significance.

## Abbreviations

CLA Conjugated linoleic acid

EX Exercise

SED Sedentary

CO Corn oil

SFO Safflower oil

pQCT peripheral quantitative computerized tomography

US United States

BMI Body mass index

BMC Bone mineral content

BMD Bone mineral density

DEXA Dual Energy x-ray absorptiometry

DFM Distal Femoral metaphysis

FD Femoral Diaphysis

PTM Proximal tibial diaphysis

TD Tibial Diaphysis

TF Tibia fibular junction

L5 Fifth lumbar vertebra

Cn B Ar Cancellous bone area

Cn BMC Cancellous bone mineral content

Cn BMD Cancellous bone mineral density

Ct B Ar Cortical bone area

Ct BMC Cortical bone mineral content

Ct BMD Cortical bone mineral density

Ct Th Cortical thickness

Peri PM Periosteal perimeter

Endo PM Endocortical perimeter

PUFA Polyunsaturated fatty acid

RANK Receptor Activated Nuclear Factor

## Competing interests

The author(s) declare that they have no competing interests.

## Authors' contributions

JB, AB and MR have made substantial contributions to acquisition of data, analysis and interpretation of data.

JB, MO and GF have been involved in drafting the manuscript, revising and given the final approval of the version to be published.
